# Beverages in Sickness

**Published:** 1855-03

**Authors:** L. A. Dugas


### DEPARTMENT OF SELECTIONS.

### REMARKS UPON THE USE OF BEVERAGES IN SICKNESS.

BY L. A. DUGAS, M. D.

Without intending for a moment to undervalue the importance of a judicious selection of the more active remedial agents in the treatment of disease, the writer nevertheless feels persuaded that much of the success of these, very often depends upon the use of proper adjuvants. The signal advantages frequently derived from the opportune administration of an enema, a foot bath, cold effusion to the head, or even a cup of tea, broth, or gruel, must have been obvious to every discerning practitioner. And yet, it is only at the bed-side that the young physician can derive much information upon the subject, as these matters of detail, cannot be or are not included in such works of general practice as are usually placed in their hands. Treatises and Lectures upon the general principles of Practice are unfortunately but little relished by students, while they read and listen with avidity to specific plans of treatment, and never fail to note down any *recipe* that may be proposed. The more violent, heroic and perturbating methods are, however, gradually giving way to milder and more judicious medication; and palliatives consequently increase in importance. The skill of the practitioner will be found to consist more in the relief of existing symptoms, than in the prescription of special formulæ learnt by rote and aimed at a name.

The use of aqueous beverages, especially in acute affections, is now so common that it cannot be a matter of indifference whether the patient partake of the one or the other of the many varieties ordinarily resorted to. The belief that the water they contain is the sole agent of value in their use, is too exclusive and prevails to too great a degree. By the ingestion of large quantities of water, and the great facility with which it is imbibed by the coats of the stomach and intestines, carried into the portal system, and from thence introduced into the general circulation, the blood is diluted and rendered less plastic, whilst the repletion of the vessels thus induced, gives increased activity to the emunctories—viz., the skin, lungs, and kidneys. The experiments of Magendie demonstrate very satisfactorily that the secretions are increased in a direct ratio with the repletion of the blood-vessels, and vice versa; that absorption is promoted in proportion to the diminution of the circulating mass.

While, therefore, in the treatment of acute diseases, which are general inflammatory, copious beverages are usually found to be useful, by diminishing the plasticity of the blood, and promoting the elimination of noxious or effete principles, their propriety is very questionable when it becomes necessary to favor absorption, as is frequently the case in chronic engorgements, serious effusions, or other deposits. When venesection is practiced, the volume of blood abstracted is very soon replaced by water; whereas by witholding such beverage, the partial vacuum of the vessels bring into them the circumjacent fluids with whatever disintegrated matters they may hold in solution. Thus it is that we may satisfactorily account for the agency of depletion and abstinence in the promotion of absorption. Yet it cannot be a matter of indifference whether the drink be acid or alkaline, stimulating sedative, mucilaginous or acrid, laxative or astringent, nutritious or not. We resort daily to beverages which, in addition to the diluent property of water, unquestionably present one or more of the peculiarities just referred to; and we should endeavor to select such as may be best adapted to each particular case. A brief enumeration of some of those in common use, and an appreciation of their peculiarities, may enable us to present our views more forcibly. They may be advantageously arranged under distinct heads, indicative of their most prominent properties.

DILUENTS.—Of all beverages, water, at the ordinary temperature of spring or well water, will be generally found the most agreeable, as well as the best, when the desired effect be simply to allay thirst or to dilute the blood. Indeed, the cravings of nature so strongly indicate the propriety of cold water as a beverage, in the fevers of our climate, that one cannot look back without a sense of horror upon the time when patients were pertinaciously denied this luxury, notwithstanding their heart-rending entreaties; when they were compelled to linger through long attacks of sickness, with parched lips and cracked tongue, lest a sip of cold water might perchance disagree with the stomach, check the perspiration, or expose them to mercurial salivation! In no particular has modern practice displayed more good sense and humanity, unless it be in the abolition of chains and the lash in the treatment of insanity, than in allowing the sick the free use of cold drinks, especially in Southern fevers. A draught of good cold water will often act like a charm, quieting the stomach, and inducing copious excretions from the skin, kidneys and lungs.

The facility with which ice is now procured in most of our towns, has led to the very free use of iced water; and, however grateful and beneficial this may be in many cases, there are circumstances in which the propriety of its use is at least questionable. In irritability of the stomach, with or without phlogosis of this viscus, iced water very generally gives relief; but in affections of the bowels, we think it decidedly objectionable. In both diarrhœa and dysentery, its bad effects are almost immediately marked by the supervention of pain and a desire to go to stool. It should also be avoided in all colicky affections, whether partaking of the nature of obstructions, of spasms or of flatulency. In bowel affections we always give the preference to warm or hot drinks. According to our bed-side observations, iced beverages should be also avoided in pulmonary diseases, and in affections of the head. We have frequently found them to induce paroxysms of coughing and dpspnœa in lung complaints, as well as pain and cerebral disturbance in affections of the brain, while tepid or warm drinks do not produce such effects. The rationale of such consequences is so evident as scarcely to need an explanation. The principle is here the same as that upon which we account for the injuries resulting from the exposure of one part of the body to cold when another part is predisposed to or actually suffering from inflammation. No one would think of plunging in iced water the feet of a patient laboring under affections of the bowels, thorax, or head; nor should the stomach be filled with iced water under such circumstances, although this organ may be benefitted by cold applications of the kind to its own surface when this is affected. The same remarks may be applied to acute affections of the skin, and old women are therefore not wrong in objecting to iced drinks in scarlatina, measles, and small-pox, however much they may err in insisting upon keeping the body excessively warm.

In the *cold* stage of our fevers we think warm drinks preferable to cold ones. They hasten the termination of the chill and bring on perspiration much sooner; and though they may be more apt to induce emesis, the very efforts to vomit materially determine the circulation to the surface, and consequently abridge the cold stage.

Demlucents.—Under this head we may place all the mucilaginous infusions, as those of Flaxseed, Slippery-elm bark, Prickly-pear, Bene leaves, Gum arabic, &c. These are nothing more than diluents in combination with bland materials. They are regarded as especially appropriate in irritations, more or less intense, of the alimentary passages, of the respiratory organs, and of the urinary apparatus. Their use has been so long sanctioned by the profession, that it is not without some hesitation that we intimate a doubt as to their real value, or rather as to their superiority over mere diluents. It can hardly be presumed that the gummy or mucilaginous materials they contain, pass into the circulation unchanged, or without previously undergoing the digestive process. They cannot therefore be viewed as bland applications to any other than the surfaces of the digestive tube. Yet they are continually prescribed as though they were destined to reach unchanged, the mucous surfaces of the lungs and urinary organs. We must confess that we have ourselves been so much in the habit of prescribing infusions of Slippery-elm and Prickly-pear in affections of the kidneys, bladder and urethra, that we should dislike to abandon them, however much we may be led by theory to doubt their intrinsic efficacy and to attribute the relief to the water and other medicinal agents with which they are administered. We must also say that we, have never perceived any advantage in the use of demulcents, as such in pulmonary diseases,—and that we really consider the one in most common use (flaxseed tea) often injurious, in consequence of the rancidity of the seed usually obtained from the shops, and the indigestibility of the infusion when made very mucilaginous, to say nothing of the unpleasantness of the dose. The other demulcents can be so readily procured in a fresh state, and are so much more agreeable, that we see no good reason for the very general use made of flaxseed tea.

The Aromatic beverages are infusions of mint, balm, sage, catnip, sassafras, &c. Their chief merit consists in being generally palatable and therefore well received by the stomach. In many instances they will relieve nausea, when this unpleasant symptom would be aggravated by demulcents. They are also decidedly anti-septic, preventing the evolution of gas by averting the tendency to fermentation, and improving the general tone of the digestive organs, without exerting injurious stimulation of the general system. They are particularly well adapted to typhoid fevers and diseases of similar character.

Catnip tea is a favorite prescription of mothers for crying babes, under the impression that the cries always indicate the existence of colic, and that catnip is a specific for this. It cannot be denied that the little creatures very frequently become quieted and go to sleep shortly after partaking freely of the well sweetened tea; but whether this effect is to be attributed to relief from colic, to some anodyne or soporific property of the tea, or simply to the fact that this operates as a substitute for the nourishment the child required, remains to be determined.

Sassafras tea is not unfrequently used in the South as a substitute for Coffee and Hyson tea, and is certainly more palatable than either of these, when as wretchedly prepared as they are in many families. Sassafras has been long supposed to possess alterative properties, and has therefore entered into the composition of most of the so-called Diet Drinks. As we do not, however, profess to understand the true meaning of the term *alterative*, as used technically, and that we consider the Diet Drinks in common use, as mere tonics or restoratives of the general stamina, we presume that

Sasafras exerts a beneficial influence upon the digestive organs. And, yet, it is difficult to determine the origin of a prejudice which exists in the minds of many of our people against its habitual use, in consequence of its supposed tendency to the production of intermittent fever. This prejudice is so general in Georgia, that it is supposed to have contributed largely, some years ago, to the defeat of a candidate for the gubernatorial chair, who had in Congress urged an increase of the duty upon tea and coffee, adding that if the enhanced price of these articles proved onerous to some, they might drink sassafras tea. The good people proudly refused to vote for any man who was willing to see them all take the ague and fever, merely for the sake of filling the National Treasury! We believe the prejudice to be unfounded—but would like to know if any *facts* can be adduced in support of it.

ASTRINGENTS.—The only beverages in common use in diease which possess any astringency, are the green and black table teas and the sage tea. This effect is, however, so slight as to be unimportant in general.

LAXATIVES.—We may class as such the infusions of Tamarinds, of dried apples, of dried peaches, of raisins, and of cream of tartar; to which may be added Saratoga water. These are all more or less grateful, and remarkably well adapted to a large class of our diseases, in which the intestines are disposed to be torpid. Those possessed of acidity promote an abundant secretion of bile as well as of gastro-intestinal fluids; hence their common use in warm climates.

ACIDS.—Lemonade and orangeade are such general favorites in diseases of tropical climates, that they are in some of the West India islands, considered as the most important medication in all affections implicating the hepatic secretion. As an anti-bilious remedy, lemonade is held in an equally high esteem by the Creoles as calomel is by the English, and those who borrow their views. Lemonade, besides being exceedingly grateful to the palate, is highly promotive of the mucous, hepatic, renal and cutaneous secretions. The free flow of salivary fluids excited by its contact with the mucous surface of the mouth and the orifices of the ducts that open upon it, will give some idea of its effect upon the gastro-intestinal surfaces and the glands whose ducts terminate in them. The capillary circulation of these mucous membranes and glandular structures, must therefore be much relieved of congestion, if any exist. But besides this local action, lemonade doubtless penetrates the general circulation by imbibition, and is carried to the kidneys and skin, whose secretions it manifestly increases. If the fluids of the system are alkaline, this is changed and they become acid by the free use of this beverage. Producing such decided local and general effects, it would seem more proper to class lemonade among the potent agents of the materia medica, than among the mere adjuvants. We feel satisfied that the therapeutic value of lemonade, in the treatment of our fevers, from the simple intermittent to the dreaded yellow fever, has not been fully appreciated by those who indite most of the books upon our shelves—the British, and our Northern brethren.

ANTACIDS.—There are states of the system in which Antacids may be eminently useful, especially if taken largely diluted or in the form of beverages. The officinal lime water, or small quantities of Bicarbonate of Soda, or of carbonate of Potass, may be thus used with plain water. The well water of blue limestone districts is sometimes of great advantage to dyspeptics. A very common error prevails with the non-professional public, who believe that soda enters into the composition of the beverage vended in our cities under the name of "Soda Water," which is nothing but water strongly impregnated with carbonic acid gas, and without any alkaline properties. The name of Soda Water had its origin in the fact that the carbonic acid gas was formerly obtained for the purpose by the action of acids upon the carbonate of soda, whereas it is now usually derived from marble or some other carbonate of lime. By the addition, however, of a little bi-carbonate of soda to this aerated water, a very pleasant and useful antacid beverage may be made.

SEDATIVES.—During the prevalence of the Broussaisin doctrine, which regarded nearly all diseases as abnormal irritations or inflammations, sedatives were eagerly sought after, in the vain hope that they would prove to be of general applicability. The distinguished French reformer, however, refused to acknowledge as such any other articles than Prussic acid and Asparagine. We may perhaps, then be excused for placing under the head of sedatives the infusions of the leaves of the Orange tree, the Lemon tree, and the Peach tree, all of which we believe contain more or less Prussic acid. Be this as it may, there is no doubt that they are exceedingly valuable beverages in our autumnal fevers. The orange-leaf tea is remarkably palatable to most persons, and in addition to being a good diluent, diaphoretic and diuretic, has a soothing effect that can scarcely be appreciated by one who has not realized it in his own person. To secure its full influence, it should be taken freely when hot, and just made, (by pouring boiling water upon the fresh leaves,) for it very soon deteriorates and becomes insipid. In the nervous affections of females, and especially in nervous head-aches, it often acts like a charm. The French make great use of the distilled orange flower water, a tea-spoonful of which they add to a glass of sweetened water;—but we think the orange-leaf tea equally valuable, and this is within the reach of every one who has a garden, as the orange tree grows finely in this region of country, and with less trouble than is required to keep the usual supply of balm, sage, &c.

The infusion of the Peach tree leaves is peculiarly useful in cases of irritable stomach, whether occurring in our fevers or after a debauch. In such cases, however, it should be made strong and given in small quantities at a time; say a table-spoonful or two, frequently repeated. In cases of hooping-cough, if given freely three or four times a day, it tends materially to lessen the violence of the paroxysms and the duration of the disease. We took occasion many years ago to allude to this use of it, and to recommend it in plantation practice, as safe and valuable.

The last class of beverages to which we shall allude, comprehends those in which NUTRITIOUS elements are added to the diluent. The most common are water holding in solution Gum Arabic, Sugar, and the various syrups, and teas made of toasted bread, rice, barley, &c. The value and applicability of these beverages are so evident, that we mention them merely for the purpose of completing the subject. Indeed we have extended our remarks so much more than we had intended when the theme first presented itself to our mind, that we now entertain serious apprehensions that the reader will be poorly repaid for the trouble of perusing them. We would accordingly withold them from our pages, were it not that we still feel that the subject is one entitled to more attention than it has heretofore received, and that the imperfections of this hasty paper may induce others to do better.—*Southern Med. and Surg. Journal.*

